# *KRIT1* Gene in Patients with Cerebral Cavernous Malformations: Clinical Features and Molecular Characterization of Novel Variants

**DOI:** 10.1007/s12031-021-01814-w

**Published:** 2021-03-02

**Authors:** Claudia Ricci, Alfonso Cerase, Giulia Riolo, Giuditta Manasse, Stefania Battistini

**Affiliations:** 1 Department of Medical, Surgical and Neurological Sciences, University of Siena, Siena, Italy; 2 Neuroimaging Unit – Diagnostic and Functional Neuroradiology, Department of Neurological and Motor Sciences, Azienda ospedaliero-universitaria Senese University Hospital, Siena, Italy

**Keywords:** CCM, *KRIT1* gene, Novel variants, De novo mutation, Functional studies, Cutaneous angioma

## Abstract

Cerebral cavernous malformations (CCMs) are vascular malformations that may result in headaches, seizures, focal neurological deficits, and hemorrhage. CCMs occur sporadically (80%) or in familial form (20%), with autosomal dominant inheritance. Among the three CCM-related genes, mutations in *KRIT1* account for 53–65% of familial cases and more than 100 different mutations have been identified so far. In the present work, we describe the clinical, neuroradiological, and genetic findings of sixteen CCM Italian patients, 13 belonging to 4 unrelated families and 3 sporadic cases. Six distinct *KRIT1* gene variants, two novel (c.1730+1_1730+3del, c.1664 C>T) and four previously described (c.966G>A, c.1255-1G>A c.1197_1200del, c.1255-1_1256del), were identified, including a possible de novo mutation. All the variants resulted in a premature stop codon. Cerebral 1.5 T magnetic resonance imaging showed multiple CCMs in all the mutation carriers for whom it was available, including sporadic cases. One patient had also cutaneous angiomas. Among the mutation carriers, symptomatic patients constituted 66% and a variable phenotypic expression was observed. Our data confirms phenotypic variability and incomplete penetrance of neurological symptoms in *KRIT1*-positive families, expands the mutational spectrum of this gene, and highlights how sporadic cases with multiple lesions need an approach similar to individuals with familial CCM.

**Supplementary Information:**

The online version contains supplementary material available at 10.1007/s12031-021-01814-w.

## Introduction

Cerebral cavernous malformations (CCMs-OMIM#116860) are vascular malformations characterized by abnormally dilated capillaries packed in sinusoids, without involvement of brain parenchyma, mainly located within the central nervous system (Denier et al. 2004; Riant et al. 2010). They result in a variety of clinical manifestations, including recurrent headaches, seizures, focal neurological deficit, and hemorrhage (Akers et al. 2017). Clinical onset is usually within the second to fourth decade of life, but symptoms can start also in early infancy or in old age (Riant et al. 2010). CCM patients are asymptomatic in up to 50% of cases. CCMs, whose prevalence in the general population is estimated to be 0.1–0.5%, may occur sporadically (80%) or in familial context (20%). Sporadic cases usually present with single cerebral lesions, while familial cases are characterized by multiple CCMs and autosomal dominant inheritance with incomplete penetrance (Akers et al. 2017).

Cerebral cavernous malformations are associated with mutations in three genes: *CCM1/KRIT1* (krev interaction trapped-1) (MIM#604214), *CCM2/MGC4607* (malcavernin) (MIM#607929), and *CCM3/PDCD10* (programmed cell death 10) (MIM#609118) (Riant et al. 2010). *KRIT1* gene mutations account for 53–65% of familial cases, and more than 100 different mutations have been identified so far (Akers et al. 2017). The majority of them are ‘loss of function’ mutations, including nonsense, frameshift, and splice site mutations, leading to a premature termination codon, or large deletions (Riant et al. 2010). Patients carrying *KRIT1* gene mutations seem to have milder hemorrhagic manifestations, but exhibit more frequently seizures as well as extra-neurological manifestations, such as cutaneous vascular malformations (Sirvente et al. 2009).

In this work, we report the clinical, neuroradiological, and genetic findings of 16 CCM Italian patients, 13 belonging to 4 unrelated families and 3 sporadic cases, carrying mutations in *KRIT1* gene, to further characterize the clinical features of CCM patients with *KRIT1* mutations and expand the mutational spectrum of this gene.

## Patients and Methods

### Patients

Seven unrelated, clinically affected CCM probands were consecutively enrolled on the basis of one of the two following criteria: each proband had at least one affected relative and/or multiple CCMs. Diagnosis was based on cerebral magnetic resonance imaging (MRI) findings. Detailed clinical and cerebral neuroradiological information was collected for all probands through direct interview and review of medical records and neuroradiological images. Clinical assessment focused on the occurrence of seizures, cerebral hemorrhage, focal neurological symptoms, and headache. Individuals with cerebral neuroradiological evidence of multiple CCMs but with negative or unavailable family history were classified as apparently sporadic cases, those with at least one affected relative as familial cases. In the last case, the study was extended to the available at-risk relatives. Written informed consent for clinical, neuroradiological, and genetic investigations was obtained from all subjects enrolled in the study.

Neuroradiological findings were reviewed by an expert neuroradiologist. Cerebral MRIs were performed on a 1.5 T magnet. Spin-echo or fast/turbo spin-echo T1- and T2-weighted, T2*-weighted, and susceptibility-weighted axial images were obtained in all the patients. CCMs were classified according to Zabramski classification system (Zabramski et al. 1994) and subsequent modifications (Bulut et al. 2014)*.* Spinal cord MRI, when available, was performed and included T1- and T2-weighted sagittal and axial images. For three patients, only neuroradiological reports were available.

### Molecular Analysis

Genomic DNA was extracted from peripheral blood by standard procedures. All 16 coding exons of the *KRIT1* gene were amplified by PCR and sequenced using an automated sequencing system. Numbering of nucleotides is according to reference sequences NG_012964.1 and NM_194456.1.

The impact of the variants on mRNA was calculated by in silico prediction using NetGene2 server (http://www.cbs.dtu.dk/services/NetGene2) (Brunak et al. 1991). The results of this analysis were confirmed by RT-PCR, as previously described (Cavé-Riant et al. 2002).

## Results

### Clinical Features

**Fig. 1:**
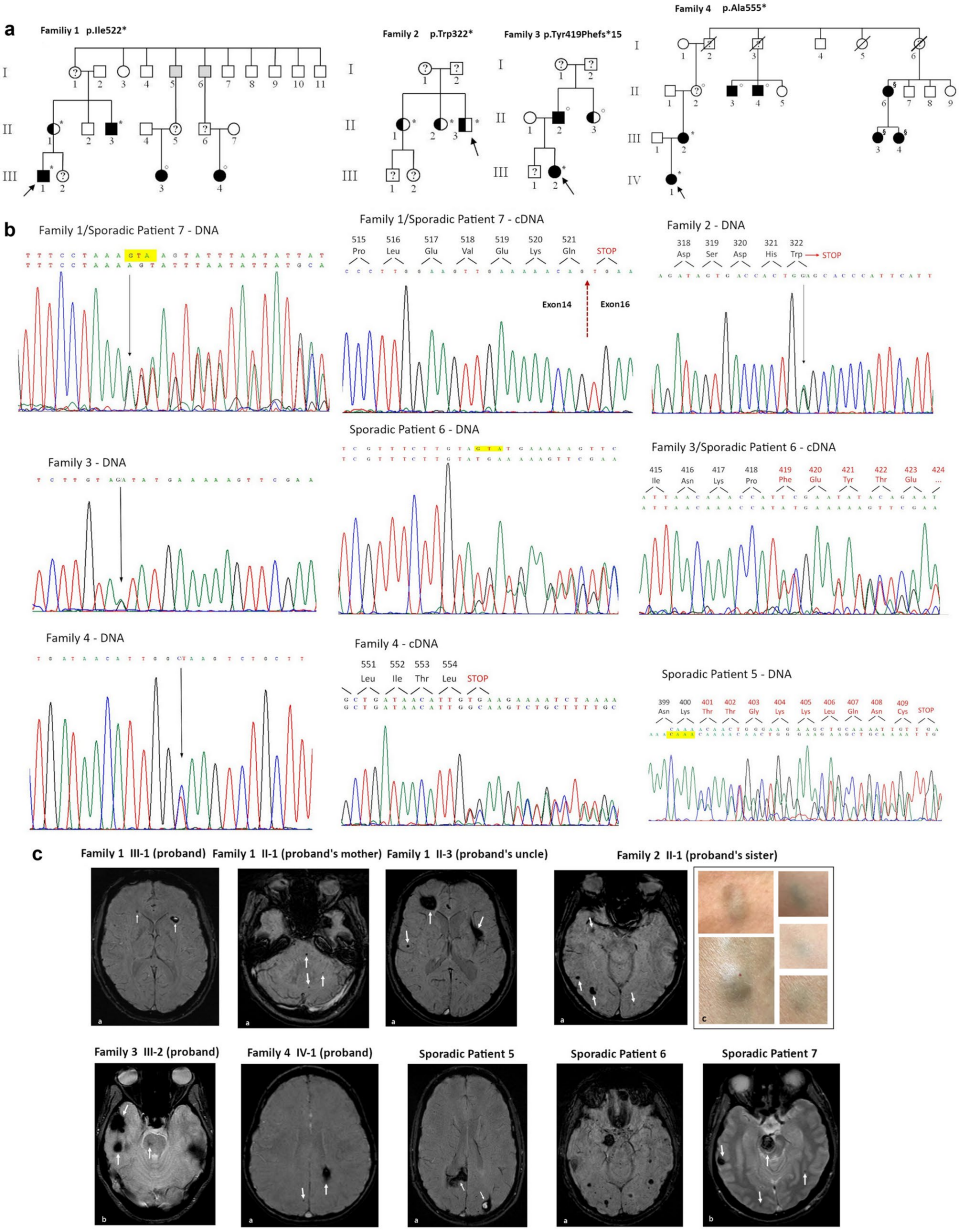
**a** Pedigrees of the four CCM families. Squares represent males and circles represent females. Full black symbols: symptomatic individuals with CCMs on MRI; white symbols: unaffected individuals; black half symbols: asymptomatic individuals with CCMs on MRI; gray symbols: individuals with epilepsy in whom MRI has not been performed; diagonal line symbols: deceased subjects; arrow: proband; “?”: MRI not performed, “*”: presence of a mutated allele; “°”: genetic analysis not carried out, “§”: mutation carriers (genetic analysis performed elsewhere). **b** Electropherograms of DNA and cDNA and corresponding amino acid sequences of variants identified in CCM patients. Arrows indicate the sites of mutation; red dotted line arrow indicates the boundary between exon 14 and exon 16. **c** Brain MRI findings in CCM patients. Susceptibility-weighted (a) and gradient echo (b) images showing multiple CCMs. Arrows indicate CCMs (in the case of sporadic patient 6, arrows are not shown due to the high number of lesions). In patient II: 1 from family 2, in addition to cerebral cavernous malformations, deep blue nodules are also noted in the subcutaneous tissue of upper limbs and neck (c)

Seven unrelated CCM patients were investigated. On the basis of the pedigree analysis, 4 of them were classified as familial and 3 as sporadic cases. In the familial cases, the study was extended to 9 at-risk relatives. Pedigrees are shown in Fig. 1a. Among the 16 CCM patients, 11 were symptomatic and 5 were symptom-free. The mean patients’ age at symptoms onset was 26.0 ± 17.9 years (range: 7 months to 53 years). Seizures were the first clinical manifestation in 8 patients (73%); cerebral haemorrhage occurred in 1 patient (9%), as well as focal neurological deficit and headache.

Regarding the asymptomatic patients, one patient (family 2, subject II-3) was admitted to the hospital at age of 39 for an ischemic stroke caused by a paradoxical embolism due to patent foramen ovale and concomitant occurrence of a deep vein thrombosis. A cerebral MRI showed multiple CCMs, in addition to the acute ischemic lesion. He came to our observation at the age of 54 without symptoms referable to the identified CCM lesions. The presence of multiple CCMs in the patient prompted us to investigate the available family members.

In addition to CCM lesions, in family 2, subject II-1 showed several cutaneous lesions characterized by blue nodules deeply situated in the subcutaneous tissue in the upper limbs and neck (Fig. 1c); in family 3, an hepatic lesion (proband III-2) and spinal cord cavernous malformations (SCCMs) (proband’s father II-2) were found. Spinal cord MRI was available only in other three patients (family 4: proband IV-1, patient 5 and 6) and did not show SCCMs.

A summary of the clinical features of the CCM probands harboring the *KRIT1* variants and their available relatives is given in Table 1. Cerebral MR images are shown in Fig. 1c. More detailed neuroradiological data are provided in the Electronic Supplementary Information (Online Resource 1).

**Table 1:** Summary of the clinical features and genetic data of CCM patients and their available family members

Family	Patient	Sex	Age at first observation	Age at diagnosis	Age at onset	Onset symptoms	No of lesions	Extra cerebral lesions	Nucleotide	Protein	Ref
1	III-1	M	22	17	8	Seizures	10	-	c.1730+1_1730+3del	p.Ile522*	This paper
	II-1	F	45	45	-	Asymptomatic	10	-	c.1730+1_1730+3del	p.Ile522*	
	II-3	M	46	45	45	Seizures	14	-	c.1730+1_1730+3del	p.Ile522*	
	III-3	F	NA	9	NA	Seizures	NA	NA	NA	NA	
	III-4	F	NA	17	NA	Seizures	NA	NA	NA	NA	
2	II-3	M	54	39	-	Asymptomatic	7	-	c.966G>A	p.(Trp322*)	9
	II-1	F	47	47	-	Asymptomatic	18	Cutaneous angioma	c.966G>A	p.(Trp322*)	
	II-2	F	55	52	-	Asymptomatic	2	-	c.966G>A	p.(Trp322*)	
3	III-2	F	29	22	22	Seizures	8	Hepatic angioma	c.1255-1G>A	p.Tyr419Phefs*15	10
	II-2	M	55	53	53	Lower left limb paresthesia	> 20	Spinal cavernous angioma	NA	NA	
	II-3	F	64	64	-	Asymptomatic	NA	-	NA	NA	
4	IV-1	F	2	2	7 months	Seizures	6	-	c.1664 C>T	p.Ala555*	This paper
	III-2	F	26	25	25	Headache	NA	-	c.1664 C>T	p.Ala555*	
Sporadic	5	M	33	9	9	Seizures	11	-	c.1197_1200del	p.(Gln401Thrfs*10)	13,14
Sporadic	6	M	55	55	33	Seizures	> 45	-	c.1255-1_1256del	p.Tyr419Phefs*15	11
Sporadic	7	M	38	38	38	Cerebral haemorrhage	21	-	c.1730+1_1730+3del	p.Ile522*	This paper

NA: not available

### Molecular Analysis

Twelve individuals were screened for *KRIT1* gene mutations. For 4 individuals, genetic testing was not available (family 1: III-3, III-4; family 3: II-2, II-3). Two novel *KRIT1* variants were identified: c.1730+1_1730+3del (family 1) and c.1664 C>T (family 4). The variant found in family 1 was also identified in the patient 7, an apparently sporadic case. Four previously described variants were found (Table 1). Segregation of the mutations with the affected phenotype was confirmed by screening the available family members.

In silico prediction and functional studies on cDNA, when available, showed that all the variants resulted in a premature stop codon (Table 2). Electropherograms of sequences obtained for DNA and cDNA are depicted in Fig. 1b.

**Table 2:** Summary of the features of the variants identified in CCM patients

Family/sporadic case	Exon	Localization	DNA	RNA	Protein	LOVD database ID	Reference
1	15	Intron	c.1730+1_1730+3del	r.1564_1730del	p.Ile522*	0000647215	This paper
2	10	Exon	c.966G>A	r. (966g>a)	p.(Trp322*)	0000647300	9
3	13	Intron	c.1255-1G>A	r.1255_1264del	p.Tyr419Phefs*15	0000647305	10
4	15	Exon	c.1664 C>T	r.1663_1730del	p.Ala555*	0000647303	This paper
5	12	Exon	c.1197_1200del	r.(1197_1200del)	p.(Gln401Thrfs*10)	0000653368	13,14
6	13	Intron/exon junction	c.1255-1_1256del	r.1255_1264del	p.Tyr419Phefs*15	0000647307	11
7	15	Intron	c.1730+1_1730+3del	r.1564_1730del	p.Ile522*	0000647215	This paper

Reference sequence: NM_194456.1. LOVD database: Leiden Open Variation Database (https://databases.lovd.nl/shared/genes

All variants have been submitted to LOVD data base (Leiden Open variation Database, https://databases.lovd.nl/shared/genes) (Table 2).

## Discussion

We identified six distinct *KRIT1* gene variants in the probands harboring multiple CCMs included in the study (Table 1): two of them were novel, whereas four had been previously reported (Nardella et al. 2018; Verlaan et al. 2002; Guarnieri et al. 2007; Zhao et al. 2011; Yang et al. 2017). They included three nucleotide substitutions and three small deletions (Table 2). All the variants led to a truncated protein, confirming the stereotyped nature of *KRIT1* variants associated with CCMs.

The c.1730+1_1730+3del deletion in exon 15 (family 1 and sporadic patient 7), not previously reported in the literature, leads to the removal of the GT nucleotides at the beginning of intron 15–16, a sequence that drives the intron recognition by the spliceosome. Functional studies on patients’ cDNA confirmed the total loss of exon 15 in the mRNA, creating a premature stop codon at the beginning of the exon 16, with the formation of a protein of 521 amino acids (p.Ile522*).

The exonic substitution c.966G>A in exon 10 (family 2), previously reported (Nardella et al. 2018), led to a nonsense codon and resulted in the predicted truncated protein p.(Trp322*). In this family, all the mutation carriers did not display neurological symptoms, despite multiple CCMs on cerebral MRI (Online Resource 1). In addition to CCM lesions, one of the proband’s sister (II-1) had several cutaneous angiomas, characterized by deep blue nodules. Cutaneous vascular malformations have been reported in 9% of patients with familial CCM, mainly associated with *KRIT1* gene variants (Sirvente et al. 2009). Among the cutaneous vascular malformation subtypes described in familial CCM, deep blue nodules account for 21% of cases (Sirvente et al. 2009).

The c.1255-1G>A substitution (family 3) and the c.1255-1_1256del deletion (sporadic patient 6) in exon 13 had been previously reported (Verlaan et al. 2002; Guarnieri et al. 2007). cDNA analysis was performed only for c.1255-1_1256del (Guarnieri et al. 2007). In the present work, functional studies displayed that both variants resulted in the same frameshift and truncated protein of 432 amino acids (p.Tyr419Phefs*15), strengthening their pathogenic role.

The exonic substitution c.1664 C>T in exon 15 (family 4), not previously described, seemed to be a missense variant (p.Ala555Val). However, in silico analysis and cDNA functional study showed that it causes the activation of a cryptic splice site and the loss of part of exon 15, modifying the reading frame and introducing a stop codon at position 555 in the protein (p.Ala555*). Interestingly, other point mutations in the *KRIT1* coding sequence do not lead to missense variants, but activate cryptic splicing sites (Riant et al. 2010; Verlaan et al. 2002; Battistini and Ricci 2020). This underlines the importance of analyzing all *KRIT1* variants, including point mutations, by in silico prediction and cDNA studies, to correctly evaluate their consequences on the protein.

Finally, the c.1197_1200del deletion in exon 12 (sporadic patient 5) had been previously described in two Chinese families (Zhao et al. 2011; Yang et al. 2017), one with 10 affected members with extreme phenotypic variability (Zhao et al. 2011). The 4-nucleotide deletion causes a shift in the reading frame, introducing a premature stop codon, and leading to a predicted protein of 411 amino acids (p.Gly401Thrfs*10).

In our *KRIT1*-positive families, a variable phenotypic expression was observed. The probands often showed a more severe clinical presentation than their relatives carrying the same mutation. In family 1, for example, the 22-year-old proband (III-1) had first seizure at the age of 8, whereas his mother (II-1), aged 45, had no symptoms despite multiple cerebral lesions. In family 4, the proband (IV-1) had seizures due to cerebral hemorrhage at the age of 7 months, whereas her mother (III-2), aged 25, had mild headache.

In addition, in family 1, we observed a decrease of age at symptom onset in subsequent generations: the proband’s maternal uncle (II-3) had a first seizure at the age of 45, while the proband presented the first seizure at the age of 8, suggesting an anticipation of the age of onset, already reported in some CCM families (Siegel et al. 1998).

Among the 12 identified mutation carriers, the proportion of symptomatic patients was 66% (8 out of 12), in agreement with literature data, confirming the incomplete penetrance of CCM-related neurological symptoms, already described in families with *KRIT1* mutations (Denier et al. 2004).

Neuroradiological penetrance of CCMs was previously considered to be complete or almost complete. Nevertheless, molecular screening of asymptomatic individuals revealed that also cerebral MRI penetrance is incomplete and age dependent (Denier et al. 2004; Battistini et al. 2007). In our study, all the mutation carriers, for whom cerebral MRI was available, showed CCM lesions, suggesting a complete neuroradiological penetrance, that, however, may be related to the small number of patients investigated.

In addition to multiple CCM lesions, spinal cord cavernous malformations (SCCMs) were found in family 3, in the proband’s father (II-2), aged 59. Spinal cord MRI was available only in other 3 patients, including the proband of family 4 (IV-1), aged 7 months, all negative for SCCMs.

SCCMs had been considered rare. However, a recent study, conducted by a 3 T MRI, showed an estimated prevalence of SCCMs, in the familial CCM patients harboring *KRIT1* variants, higher than previously reported (approximately 70%), and correlated with patients’ age and number of cerebral cavernous lesions, suggesting that familial CCM is a progressive systemic disease affecting the entire central nervous system (Mabray et al. 2020). In our study, some limitations, including the small sample size and the MRI magnet (1.5 T) used in clinical practice, do not allow evaluating the SCCM prevalence in familial CCM patients harboring *KRIT1* mutations. Nonetheless, the very young age of family 4 proband does not rule out that SCCM lesions might develop later.

In the present work, all the sporadic patients with *KRIT1* mutations had multiple CCMs. A study on *KRIT1* gene mutations in sporadic cases showed the presence of mutations in 29% of sporadic cases with multiple lesions, while no mutation was identified in patients with single lesions (Verlaan et al. 2004). Our findings strengthen the observation that sporadic cases with multiple CCMs need an approach similar to that used for familial cases.

Clinical and genetic data reported so far have shown that the majority of sporadic patients with multiple lesions are actually cases with mutations inherited from an asymptomatic parent or with de novo mutations (Riant et al. 2010; Labauge et al. 2007). In our study, genetic analysis in parents of sporadic cases was possible only for patient 5. The c.1197_1200del variant was not present in the parents, both asymptomatic and negative for CCM lesions on cerebral MRI. A de novo mutation may be arisen in the patient; however, an incorrect paternity attribution cannot be ruled out, since the paternity test could not be performed. De novo* KRIT1* gene mutations have been described occasionally, but their real percentage may be underestimated, because genetic analysis in parents is not always possible (Rath et al. 2017). These mutations may occur randomly at any stage of embryonic development, or at the germline level in the parents’ gametes, positively correlating with parents’ age (Goldmann et al. 2019). These aspects must be considered for a correct genetic counseling in sporadic cases carrying CCM gene mutations.

In conclusion, our data confirm the phenotypic variability of CCM patients with *KRIT1* mutations, expand the mutational spectrum of this gene, and highlight how sporadic cases with multiple lesions need an approach similar to individuals with familial CCM.

## Supplementary Information

Below is the link to the electronic supplementary material.

Supplementary file1 (DOCX 51 KB)

## Data Availability

Variant data have been submitted to LOVD data base (Leiden Open variation Database) https://databases.lovd.nl/shared/genes. The data that support the findings of this study are available on request from the corresponding author.

## References

[CR1] Akers A, Al-Shahi Salman R, Award IA, Dahlem K, Flemming K, Hart B, Kim H, Jusue-Torres I, Kondziolka D, Lee C, Morrison L, Rigamonti D, Rebeiz T, Tournier-Lasserve E, Waggoner D, Whitehead K (2017). Synopsis of guidelines for the clinical management of cerebral cavernous malformations: consensus recommendations based on systematic literature review by the angioma alliance scientific advisory board clinical experts panel. Neurosurgery.

[CR2] Battistini S, Ricci C (2020) Concern regarding classification of c.703G>A/p.Gly235Arg as a novel missense variant in KRIT1 gene. Human Mutat 41:1069–1071. 10.1002/humu.24000 10.1002/humu.24000 32285596

[CR3] Battistini S, Rocchi R, Cerase A, Citterio A, Tassi L, Lando G, Patrosso MC, Galli R, Brunori P, Sgrò DL, Pitillo G, Lo Russo G, Marocchi A, Penco S (2007) Clinical, magnetic resonance imaging, and genetic study of 5 Italian families with cerebral cavernous malformation. Arch Neurol 64:843–848. 10.1001/archneur.64.6.843 10.1001/archneur.64.6.843 17562932

[CR4] Brunak S, Engelbrecht J, Knudsen S (1991) Prediction of human mRNA donor and acceptor sites from the DNA sequence. J Mol Biol 220:49–65. 10.1016/0022-2836(91)90380-o 10.1016/0022-2836(91)90380-o 2067018

[CR5] Bulut HT, Sarica MA, Baykan AH (2014). The value of susceptibility weighted magnetic resonance imaging in evaluation of patients with familial cerebral cavernous angioma. Int J Clin Exp Med.

[CR6] Cavé-Riant F, Denier C, Labauge P, Cécillon M, Maciazek J, Joutel A, Laberge-Le Couteulx S, Tournier-Lasserve E (2002) Spectrum and expression analysis of KRIT1 mutations in 121 consecutive and unrelated patients with cerebral cavernous malformations. Eur J Hum Genet 10:733–740. 10.1038/sj.ejhg.5200870 10.1038/sj.ejhg.5200870 12404106

[CR7] Denier C, Labauge P, Brunereau L, Cavé-Riant F, Marchelli F, Arnoult M, Cecillon M, Maciazek J, Joutel A, Tournier-Lasserve E (2004) Clinical features of cerebral cavernous malformations patients with KRIT1 mutations. Ann Neurol 55:213–220. 10.1002/ana.10804 10.1002/ana.10804 14755725

[CR8] Goldmann JM, Veltman JA, Gilissen C (2019) De novo mutations reflect development and aging of the human germline. Trends Genet 35:828–839. 10.1016/j.tig.2019.08.005 10.1016/j.tig.2019.08.005 31610893

[CR9] Guarnieri V, Muscarella LA, Amoroso R, Quattrone A, Abate ME, Coco M, Catapano D, D'Angelo VA, Zelante L, D'Agruma L (2007) Identification of two novel mutations and of a novel critical region in the KRIT1 gene. Neurogenetics 8:29–37. 10.1007/s10048-006-0056-y 10.1007/s10048-006-0056-y 17043900

[CR10] Labauge P, Denier C, Bergametti F, Tournier-Lasserve E (2007) Genetics of cavernous angiomas. Lancet Neurol 6:237–244. 10.1016/S1474-4422(07)70053-4 10.1016/S1474-4422(07)70053-4 17303530

[CR11] Mabray MC, Starcevich J, Hallstrom J, Robinson M, Bartlett M, Nelson J, Zafar A, Kim H, Morrison L, Hart BL (2020) High prevalence of spinal cord cavernous Malformations in the familial cerebral cavernous malformations type 1 cohort. AJNR Am J Neuroradiol 41:1126–1130. 10.3174/ajnr.A6584 10.3174/ajnr.A6584 PMC7342759 32467184

[CR12] Nardella G, Visci G, Guarnieri V, Castellana S, Biagini T, Bisceglia L, Palumbo O, Trivisano M, Vaira C, Scerrati M, Debrasi D, D'Angelo V, Carella M, Merla G, Mazza T, Castori M, D'Agruma L, Fusco C (2018) A single-center study on 140 patients with cerebral cavernous malformations: 28 new pathogenic variants and functional characterization of a PDCD10 large deletion. Human Mutat 39:1885–1900. 10.1002/humu.23629 10.1002/humu.23629 30161288

[CR13] Rath M, Jenssen SE, Schwefel K, Spiegler S, Kleimeier D, Sperling C, Kaderali L, Felbor U (2017) High-throughput sequencing of the entire genomic regions of CCM1/KRIT1, CCM2 and CCM3/PDCD10 to search for pathogenic deep-intronic splice mutations in cerebral cavernous malformations. Eur J Med Genet 60:479–484. 10.1016/j.ejmg.2017.06.007 10.1016/j.ejmg.2017.06.007 28645800

[CR14] Riant F, Bergametti F, Ayrignac X, Boulday G, Tournier-Lasserve E (2010) Recent insights into cerebral cavernous malformations: the molecular genetics of CCM. FEBS J 277:1070–1075. 10.1111/j.1742-4658.2009.07535.x 10.1111/j.1742-4658.2009.07535.x 20096038

[CR15] Siegel AM, Andermann E, Badhwar A, Rouleau GA, Wolford GL, Andermann F, Hess K (1998) Anticipation in familial cavernous angioma: a study of 52 families from international familial cavernous angioma study. IFCAS Group. Lancet 352:1676–1677. 10.1016/s0140-6736(05)61447-x 10.1016/s0140-6736(05)61447-x 9853443

[CR16] Sirvente J, Enjolras O, Wassef M, Tournier-Lasserve E, Labauge P (2009) Frequency and phenotypes of cutaneous vascular malformations in a consecutive series of 417 patients with familial cerebral cavernous malformations. J Eur Acad Dermatol Venereol 23:1066–1072. 10.1111/j.1468-3083.2009.03263.x 10.1111/j.1468-3083.2009.03263.x 19453802

[CR17] Verlaan DJ, Laurent SB, Sure U, Bertalanffy H, Andermann E, Andermann F, Rouleau GA, Siegel AM (2004) CCM1 mutation screen of sporadic cases with cerebral cavernous malformations. Neurology 62:1213–1215. 10.1212/01.wnl.0000118299.55857.bb 10.1212/01.wnl.0000118299.55857.bb 15079030

[CR18] Verlaan DJ, Siegel AM, Rouleau GA (2002) Krit1 missense mutations lead to splicing errors in cerebral cavernous malformation. Am J Hum Genet 70:1564–1567. 10.1086/340604 10.1086/340604 PMC379143 11941540

[CR19] Yang C, Zhao J, Wu B, Zhong H, Li Y, Xu Y (2017) Identification of a novel deletion mutation (c.1780delG) and a novel splice-site mutation (c.1412–1G>A) in the CCM1/KRIT1 gene associated with familial cerebral cavernous malformation in the Chinese population. J Mol Neurosci 61:8–15. 10.1007/s12031-016-0836-2 10.1007/s12031-016-0836-2 27649701

[CR20] Zabramski JM, Wascher TM, Spetzler RF, Johnson B, Golfinos J, Drayer BP, Brown B, Rigamonti D, Brown G (1994) The natural history of familial cavernous malformations: results of an ongoing study. J Neurosurg 80:422–432. 10.3171/jns.1994.80.3.0422 10.3171/jns.1994.80.3.0422 8113854

[CR21] Zhao Y, Xie L, Li P, Song J, Qu T, Fan W, Chen H, Chen D, Lu D, Zhou L, Mao Y (2011) A novel CCM1 gene mutation causes cerebral cavernous malformation in a Chinese family. J Clin Neurosci 18:61–65. 10.1016/j.jocn.2010.04.051 10.1016/j.jocn.2010.04.051 20884211

