# Supplementary material for: KRIT1 Gene in Patients with Cerebral Cavernous Malformations: Clinical Features and Molecular Characterization of Novel Variants
Source: J Mol Neurosci. 2021 Mar 2;71(9):1876–83. doi: 10.1007/s12031-021-01814-w (PMC8421287; doi:10.1007/s12031-021-01814-w)
Supplement: Supplementary file 1 — Supplementary file1 (DOCX 51 KB) [file 12031_2021_1814_MOESM1_ESM.doc]

| **Family** | **Patients** | **Sex** | **Age**  **at last MRI** | **CNS CCMs**  **number** | **CNS CCMs**  **site / type** | | **Spinal cord**  **MRI** |
| --- | --- | --- | --- | --- | --- | --- | --- |
| 1 | **III-1** | M | 26 | 10 | rU/I (II, 4 years before), rT/III, lIns/I (II, 4 years before), lF/III, lO/V, rPa/III atypical, lCe/V°, rCe/IV°, rCe/IV°, lT/V°  ° newly appeared at 4-year follow-up | NP | |
|  | II-1 | F | 45 | 10 | rCe/IV, lCe/III, lCe/V, lPo/III atypical, lPo/V, lT/II, rT/III, lPa/II, rCS/IV-V, lF/III atypical | NP | |
|  | II-3 | M | 50 | 14 | rF/II, lIns/II, lS/IV, rF/IV, rF/IV, lF/IV, rT/IV, RPo/V, cP/V, PM/V, rPo/III atypical°, lO/IV°, lT/II°, rO/III°  ° newly appeared at 4-year follow-up | NP | |
| 2 | **II-3** | M | 39 | 7 | rCS, lF, lP, lT, rT* | NP | |
|  | II-1 | F | 52 | 18 | rCe/III atypical, rPo/IV, rPo/IV, rT/II, rT/IV, rT/III, lT/IV, lT/IV  rT-O/III atypical, lTh/IV, AC/IV, lO/IV, rF/II, rF/II (IV, five years before), lF/IV, rPa/IV, lP/II, rF/IV°  ° newly appeared at 3-year follow-up | NP | |
|  | II-2 | F | 52 | 2 | pPo, rO** | NP | |
| 3 | **III-2** | F | 29 | 8 | rT/ II, rFo/IV, rP/II, rSC/IV, lS/IV, lO/IV, lF/V, lFo/V | NP | |
|  | II-2 | M | 53 | >20 | Larger one in the lCS* | C3-D1 and D3 levels | |
| 4 | **IV-1** | F | 6 | 6 | rT/IV, lPO/IV, rCa/IV, lPa/II, lCS/IV, lAC/V°  ° newly appeared at 4-year follow-up | No CCMs | |
| 5 | **TP** | M | 33 | 11 | rCe/I°, lCe/I°, rT/III°, rT/IV°, rO/V°, lT/III°, lF/IV, lO /IV, lO /I (II, 16 months before), rPa/II°, rF/IV  ° newly appeared at 14-year follow-up | No CCMs | |
| 6 | **DV** | M | 55 | >45 | B, lCe, rCe, lC, rC/IV and V, rT/I, rT/II | No CCMs | |
| 7 | **VM** | M | 38 | 21 | rPo/III, lPo/IV, P-M/I, rCe/IV, lCe/IV, lCe/IV, rO/IV, lT/IV, lT/III atypical, rT/III, rT/III, lT/IV, rLN/II, lPa/II, F paramedian/ IV,  rF ant/III, rPs/III atypical, rF/IV, lPV/IV, lPa/IV, lPa/III | NP | |

**Table S1** **Summary of the neuroradiological data of CCMs patients and their affected family members**.

l: Left; r: Right; AC: Anterior capsule; B: Brainstem; C: cerebral hemisphere; Ce: cerebellar hemisphere; cPo: central pons; CS: centrum semiovale; F: Frontal; Fo: Fronto-opercolar; Ins: Insula; O: Occipital; Pa: Parietal; Po: Pons; pPo: posterior Pons; S: Callosal splenium; T: Temporal; Th: Thalamus; T-O: Temporo-occipital; U: uncus; *from MRI reports: CCMs sites and types not better reported; **from Computed Tomography (CT) report; NP: not performed.
